# Supplementary figures and images for: Conduction pathway for potassium through the Escherichia coli pump KdpFABC
Source: eLife. 2025 Nov 19;14:RP107397. doi: 10.7554/eLife.107397 (PMC12629597; doi:10.7554/eLife.107397)

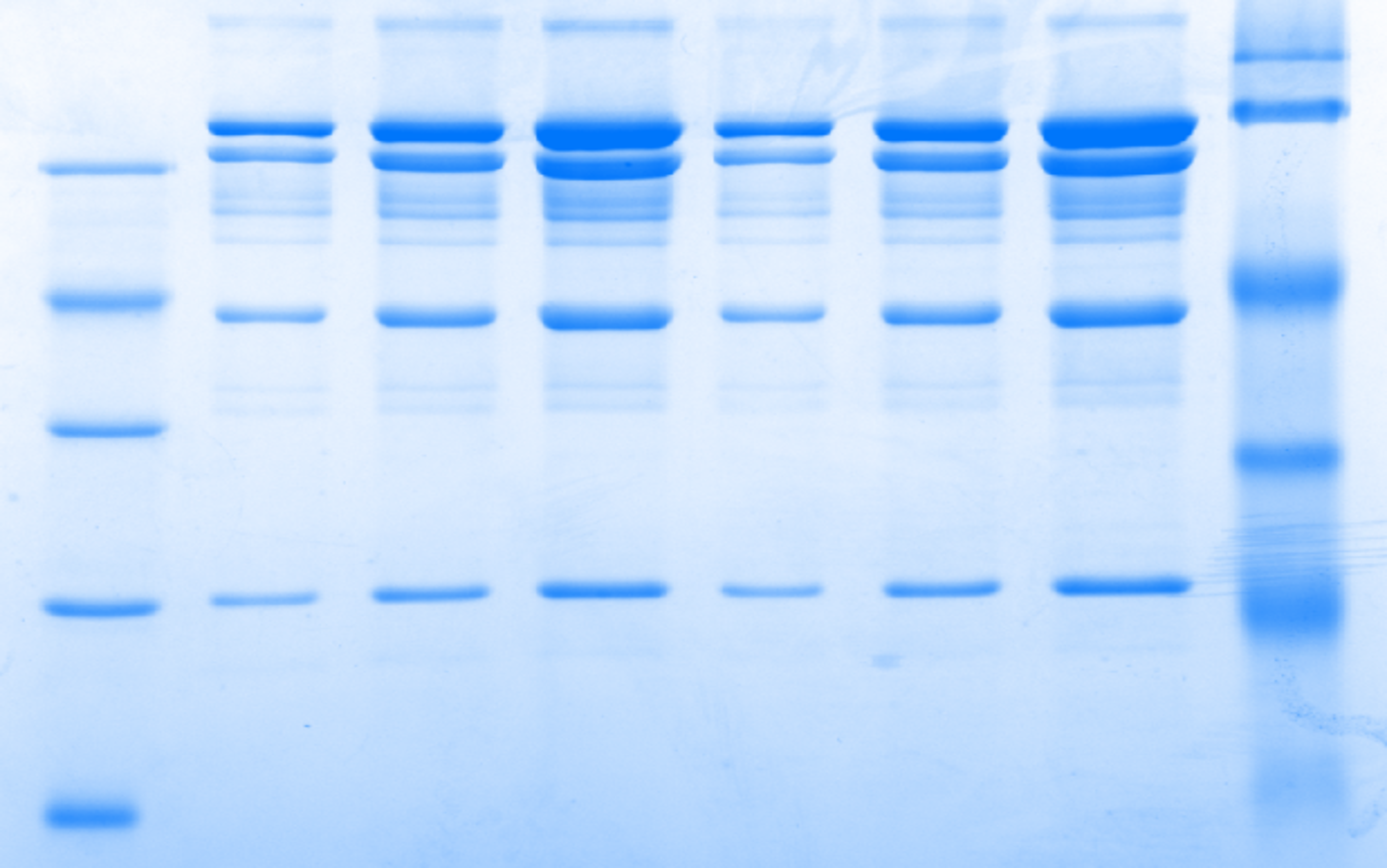

Supplement: Figure 1—figure supplement 1—source data 2. [file elife-107397-fig1-figsupp1-data2.zip › nanodisc-gel.tif]

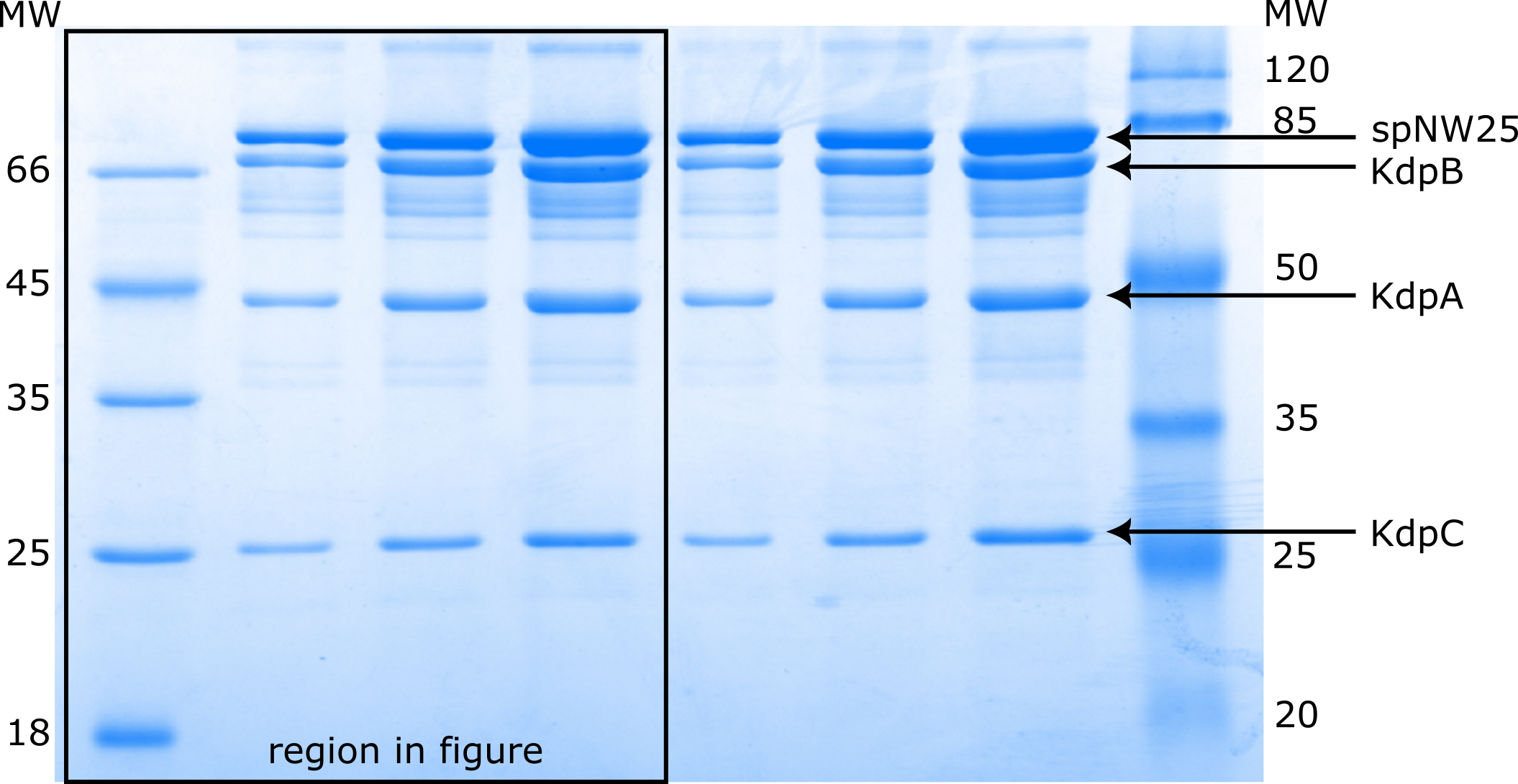

Supplement: Figure 1—figure supplement 1—source data 3. [file elife-107397-fig1-figsupp1-data3.zip › uncropped_gel_labels.tiff]
